# A Fréchet tree distance measure to compare phylogeographic spread paths across trees

**DOI:** 10.1038/s41598-018-35421-4

**Published:** 2018-11-19

**Authors:** Susanne Reimering, Sebastian Muñoz, Alice C. McHardy

**Affiliations:** 1 Department for Computational Biology of Infection Research, Helmholtz Center for Infection Research, Braunschweig, Germany; 2 German Center for Infection Research (DZIF), Braunschweig, Germany

## Abstract

Phylogeographic methods reconstruct the origin and spread of taxa by inferring locations for internal nodes of the phylogenetic tree from sampling locations of genetic sequences. This is commonly applied to study pathogen outbreaks and spread. To evaluate such reconstructions, the inferred spread paths from root to leaf nodes should be compared to other methods or references. Usually, ancestral state reconstructions are evaluated by node-wise comparisons, therefore requiring the same tree topology, which is usually unknown. Here, we present a method for comparing phylogeographies across different trees inferred from the same taxa. We compare paths of locations by calculating discrete Fréchet distances. By correcting the distances by the number of paths going through a node, we define the Fréchet tree distance as a distance measure between phylogeographies. As an application, we compare phylogeographic spread patterns on trees inferred with different methods from hemagglutinin sequences of H5N1 influenza viruses, finding that both tree inference and ancestral reconstruction cause variation in phylogeographic spread that is not directly reflected by topological differences. The method is suitable for comparing phylogeographies inferred with different tree or phylogeographic inference methods to each other or to a known ground truth, thus enabling a quality assessment of such techniques.

## Introduction

Phylogeography combines phylogenetic information describing the evolutionary relationships among species or members of a population with geographic information to study migration patterns. Given known locations for the taxa assigned to the leaf nodes of a phylogenetic tree, the putative locations for the internal nodes can be inferred by ancestral character state reconstruction. This reveals putative spread paths from the root of a tree to the leaves. Using either discrete locations like cities, countries and continents^1^ or continuous locations based on longitude and latitude^2,3^, phylogeography has been applied to analyze a wide range of organisms, including viruses like influenza^1,4,5^, HIV^6^ and rabies^1,2^. These analyses give insights to the origin and spread of viral pathogens and are essential for effective disease control and surveillance. State-of-the art methods are usually based on Bayesian inference^1,2^ and evaluate different solutions by their posterior probabilities, but a direct comparison of spread patterns inferred by different methods is not commonly performed.

Ancestral character state reconstructions can be evaluated by comparison to a reference generated by simulations^7,8^, experimentally generated values^9^ or fossil records^10^. However, these methods usually require the same topology for the trees with reference states and inferred states. Nodes are compared in a pairwise manner to calculate correlations or squared deviations^7,9,10^ or to compare ancestral state distributions at single nodes^8^. In practice, however, the true tree topology likely differs from a topology inferred from genetic data. To compare different phylogeographic approaches, which may simultaneously reconstruct the tree and ancestral locations^1^, a distance measure to compare reconstructed locations between different trees is required. One possible solution used in the past is to compare the most recent common ancestor of a set of taxa^11^. This allows different tree topologies, but still results in a pairwise comparison of nodes. For phylogeographic reconstructions, it would be desirable to compare reconstructed spread routes instead of pairs of nodes and to consider geographic distances between these routes. To our knowledge, no method fulfilling all these criteria exists to this date.

For these reasons, we describe a distance measure and algorithm for comparing reconstructed phylogeographic spread patterns between different tree topologies inferred from the same set of taxa. Instead of performing pairwise comparisons between nodes we look at paths of locations from the root of the tree to the leaves, compare these using a variation of the discrete Fréchet distance and correct for the number of paths going through each node. The Fréchet distance was originally introduced to compare curves^12^, followed by a discrete variation^13^, also called coupling distance, to calculate distances between polygonal curves. The discrete Fréchet distance allows to compare between paths of different lengths while preserving the order of the points. By incorporating this distance measure into an algorithm to assess paths along phylogenetic trees, we can compare reconstructed phylogeographic spread patterns to a reference and evaluate their differences. To demonstrate the application, we inferred different phylogenetic trees for hemagglutinin (HA) sequences of avian H5N1 influenza viruses, applied parsimony-based phylogeographic inference and evaluated the influence of the tree reconstruction method on geographic spread. The results suggest that phylogenetic tree inference can have a large effect on the reconstructed spread patterns, which is not as apparent when comparing the topologies. Intriguingly, the reconstruction by neighbor joining is a clear outlier compared to the parsimony, maximum likelihood and UPGMA reconstructions, which does not reflect the underlying methodological similarities (character-based versus distance methods) and varying degrees of flexibility (e.g. UPGMA assumes a strict molecular clock; a strong restriction none of the other methods make). Phylogeographic inference using maximum likelihood confirmed that the observed variation is not only due to the use of parsimony for ancestral state reconstruction. However, individual differences varied compared to the inference using parsimony. In summary, our analysis shows that both tree inference and ancestral reconstruction influence the results of phylogeographic studies. Together with suitable references generated i.e. by combining simulations of disease transmission and simulations of sequence evolution, this distance measure can be applied to benchmark different phylogeographic reconstructions to gain a more profound understanding which methods, models or parameters work well under which conditions.

## Data and Methods

### Discrete Fréchet distance on trees

The discrete Fréchet distance compares two polygonal curves by defining a coupling between the two sets of points in a way that all points are linked, the start and end points are connected and the direction is preserved. The coupling is chosen in a way that it minimizes either the maximum distance or the sum of distances between points. This minimized distance measures the similarity between the two polygonal curves. In the following, we describe this method in detail and illustrate its extension to compare phylogeographic spread paths across trees.

**Figure 1:**
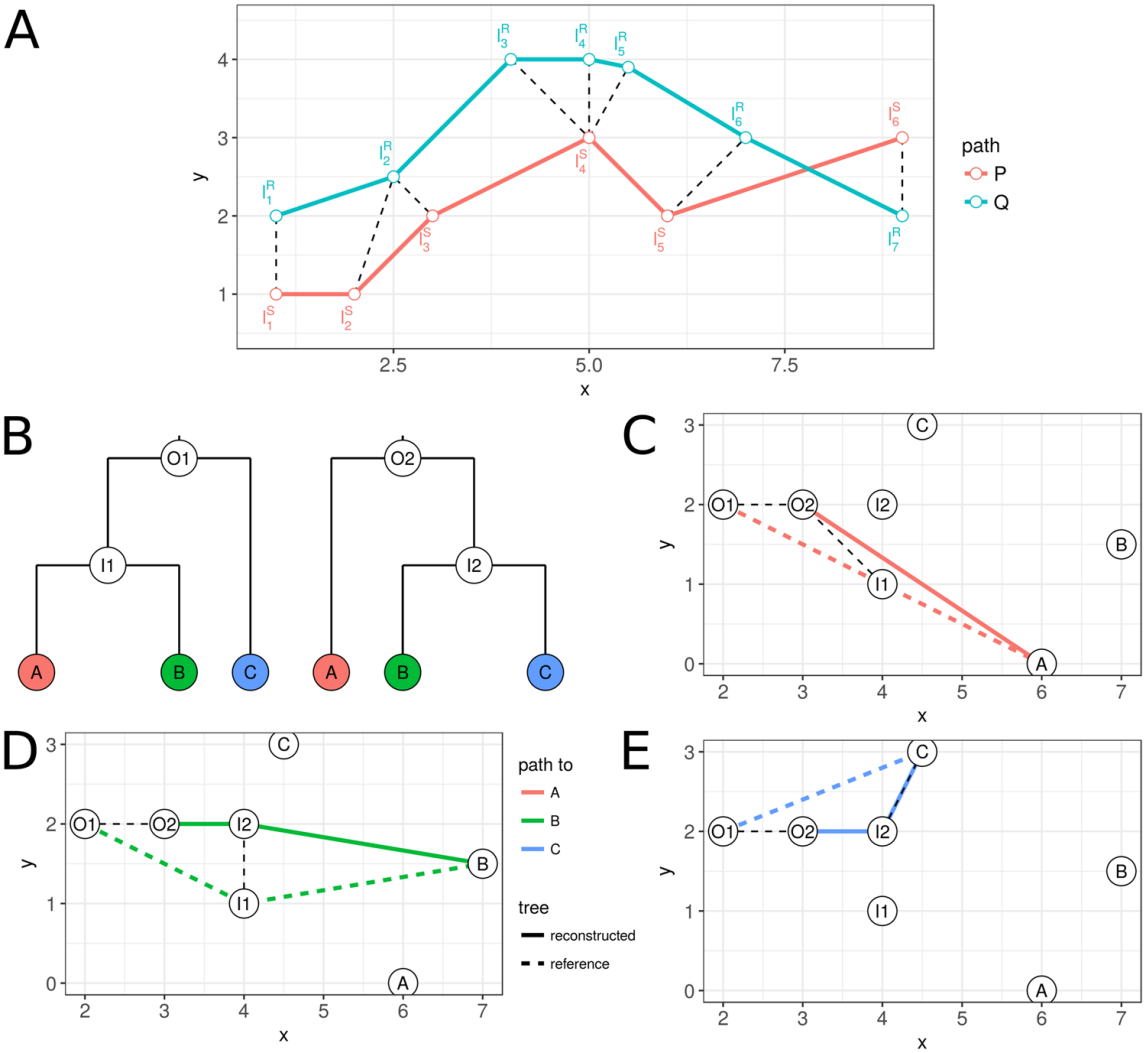
Calculation of the discrete Fréchet tree distances on two paths and on two phylogenetic trees with different topologies. (**A**) Coupling which minimizes the discrete Fréchet distance between two paths P (red) and Q (blue) with different lengths. The coupling between points is indicated by the black dashed line. (**B**) Example of two phylogenetic trees (reference on the left, reconstruction on the right) with different topologies inferred on the same taxa. Labels at the nodes indicate the locations, which are shown on a two-dimensional map in C, D and E. For each leaf node, the paths along the trees are compared. (**C**) Comparison of paths to location A. (**D**) Comparison of paths to location B. (**E**) Comparison of paths to location C. The coupling of nodes minimizing the distance between the paths is indicated by black dashed lines. For each node, these distances are summarized across all leaves. In case of the reference tree, this leads to the following calculations: $cost(O1)=3\times d(O1,O2)$, $cost(I1)=d(I1,O2)+d(I1,I2)$, $cost(A)=0$, $cost(B)=0$, $cost(C)=d(C,I2)$. These costs are then divided by the number of descendant leaves and summarized to calculate a final cost for the reference tree.

Let *S* be a phylogenetic tree with *n* leaf nodes, *m*^*s*^ internal nodes, and locations ${l}_{i}^{S}$ for all leaf and internal nodes. *R* represents a second phylogenetic tree with the same *n* leaf nodes and *m*^*R*^ internal nodes, as well as locations ${l}_{i}^{R}$ for all leaf and internal nodes. We assume that the same locations have been assigned to the *n* leaf nodes of *S* and *R*, although both the topology and the locations assigned to internal nodes can differ. Given a single leaf node, we now define the following:

Let $P=({l}_{1}^{S},\mathrm{..}.,\,{l}_{p}^{S})$ and $Q=({l}_{1}^{R},\mathrm{..}.,\,{l}_{q}^{R})$ be the sequence of locations, i.e. the geographic paths from the root node to a specific leaf node in *S* and *R*, respectively. As defined by Eiter and Mannila^13^, a coupling *L* between *P* and *Q* is a sequence

$$({l}_{{a}_{1}}^{S},{l}_{{b}_{1}}^{R}),({l}_{{a}_{2}}^{S},{l}_{{b}_{2}}^{R}),\mathrm{..}.,({l}_{{a}_{x}}^{S},{l}_{{b}_{x}}^{R})$$

of distinct pairs from *P* and *Q* with *a*_1_ = 1, *b*_1_ = 1,*a*_x_ = *p, b*_*x*_ = *q* (i.e. the start and end locations are linked). *a*_*i*+1_ is defined as either *a*_*i*+1_ = *a*_*i*_ or *a*_*i*+1_ = *a*_*i+*1_, and *b*_*i*+1_ as *b*_*i*+1_ = *b*_*i*_ or *b*_*i*+1_ = *b*_*i+*1_. This ensures that the coupling preserves the order of the nodes. Figure 1A illustrates the coupling of nodes between two paths in a two-dimensional space.

Eiter and Mannila^13^ then define the length $||L||$ of the coupling *L* as the maximum distance between the coupled nodes. As a possible variation, which we are using for the implementation on geographic paths as well, they define the length as the sum of all distances, i.e.:

$$||L||=\sum _{i=1}^{x}d({l}_{{a}_{i}}^{S},{l}_{{b}_{i}}^{R})$$

The discrete Fréchet distance between paths, denoted here as ${\delta }_{DF}$, then finds the coupling of nodes that minimizes $||L||$, i.e.:

$${\delta }_{DF}(P,Q)=min\{||L||,L\,is\,a\,coupling\,between\,P\,and\,Q\}$$

Note that by defining the length $||L||$ of the coupling as the sum of all distances (instead of the maximum), the discrete Fréchet distance is not a real metric, as the condition of triangle inequality is usually not satisfied^13^. We here use geographical distances between locations. Generally, other distance measures could also be used. Measures like effective distances, which define the distance between places by the amount of people moving between them^14^, could e.g. be used to evaluate phylogeographies inferred for pathogens mainly spreading via air travel.

To expand this measure to whole phylogenetic trees, we take each leaf node *i* in *S* and *R* and compare the paths *P*_*i*_ and *Q*_*i*_ to calculate ${\delta }_{DF}({P}_{i},{Q}_{i})$. In this step, we determine the coupling with the minimal length. To combine the distances over all leaf nodes, we then define a cost for all nodes in the tree. Let *k* be a node in *S* and *n*_*k*_ the set of leaves that are descendants of *k*. For each leaf node $i\in {n}_{k}$ the path *P*_*i*_ therefore includes ${l}_{k}^{S}$, i.e. the location assigned to *k*, and we can define

$${c}_{k}({P}_{i},\,{Q}_{i})=\{\,j|({l}_{k}^{S},\,{l}_{j}^{R})is\,a\,pair\,in\,the\,coupling\,between\,{P}_{i}\,and\,{Q}_{i}\}$$

as the set of nodes in the tree *R* that are connected to node *k* in *S*. The overall cost for *k* over all leaves and all connections is then calculated by

$$cost(k)=\sum _{i\in {n}_{k}}\sum _{j\,\in {c}_{k}({P}_{i},{Q}_{i})}d({l}_{k}^{S},{l}_{j}^{R})$$

This adds the distances of each node in all paths to an overall cost. For the cost of the whole tree, we first divide the overall cost of a node by the number of descendant leaves, i.e. the number of paths going through this node, and sum up the values for all leaf nodes *n* and internal nodes *m*^s^ in the tree *S*:

$$cost(S)=\sum _{k=1}^{n+{m}^{s}}\frac{\cos \,t(k)}{|{n}_{k}|}$$

The division by the number of paths ensures that deviations occurring close to the root, which are therefore included in many paths, have the same weight as deviations occurring further down in the tree. Moreover, differences in tree shape that influence the number of descendant leaf nodes and with that the number of paths for specific nodes, e.g. the balance of the tree, are compensated this way. The general approach is illustrated in Fig. 1B–E. For the tree *R*, the cost is calculated in an equivalent manner. The average of the costs for *S* and *R* results then in the overall distance between the two trees.

### Data, phylogenetic inference and phylogeographic reconstruction

Hemagglutinin (HA) sequences of 190 H5N1 influenza viruses were downloaded from the NCBI Influenza Database^15^. The dataset was first compiled by Wallace *et al*.^16^ and further analyzed by Lemey *et al*.^1^. In total, both studies analyzed 192 isolates, but only for 190 isolates HA sequences were available. The sequences were sampled between 1996 and 2005 from 20 different locations in Asia and Europe. HA sequences were aligned using MUSCLE^17^ and trimmed using TrimAl^18^ to remove positions of low quality with gaps in more than 80% of the sequences. Phylogenetic trees were inferred from the multiple sequence alignment using the R package phangorn^19^ with the following methods: parsimony using the Fitch algorithm, neighbor joining (NJ), UPGMA and maximum likelihood using the Jukes-Cantor (MLJC) and the general time reversible model (MLGTR). For the distance-based methods (NJ and UPGMA), distances were calculated using the Jukes-Cantor substitution model. All trees were rooted using the H5N1 influenza virus isolate A/chicken/Scotland/59 as an outgroup, which was subsequently removed from the analysis. To infer locations for internal nodes, ancestral character state reconstruction (ASR) was performed on all trees. For parsimonious ASR, the implementation of the Fitch algorithm using accelerated transformation from the phangorn package^19^ was used. ASR using maximum likelihood was conducted using the R package ape^20^.

### Calculation of geographical distances and clustering of states

The calculation of the discrete Fréchet distance between trees requires a matrix with distances between all observed locations. Here, we use geographical distances. We first inferred geographical coordinates (latitude and longitude) for each location using the geocode function of the R package ggmap^21^ and calculated geographical distances between them using the R package geosphere^22^. These geographical distances represent the shortest path (given in kilometers) between two locations on the ellipsoidal surface of the Earth.

To enable maximum likelihood ASR, it was necessary to merge the original locations to reduce the number of states. With the original locations (20 in total), both the function ancestral.pml from the R package phangorn and the ace and reconstruct functions from the ape package failed to run. We used complete linkage hierarchical clustering on the previously calculated distance matrix to summarize the locations into 10 clusters based on their geographical proximity. This analysis clustered Bangkok, Nakon Sawan, Uthai Than, Phitsanulok and Kamphaeng Phet into one cluster, Hong Kong, Guangdong, Fujian, Hunan and Guangxi into another, as well as Hebei, Henan and Shanghai into a third. All other clusters contained only one location. We denoted the new clustered locations as Thailand, Southern China and Northern China. Both the parsimony and maximum likelihood ASR reconstructions were performed on these clustered locations instead of the original ones. The distances between the clusters, as calculated by complete linkage, were subsequently used to calculate discrete Fréchet tree distances.

## Results

**Figure 2:**
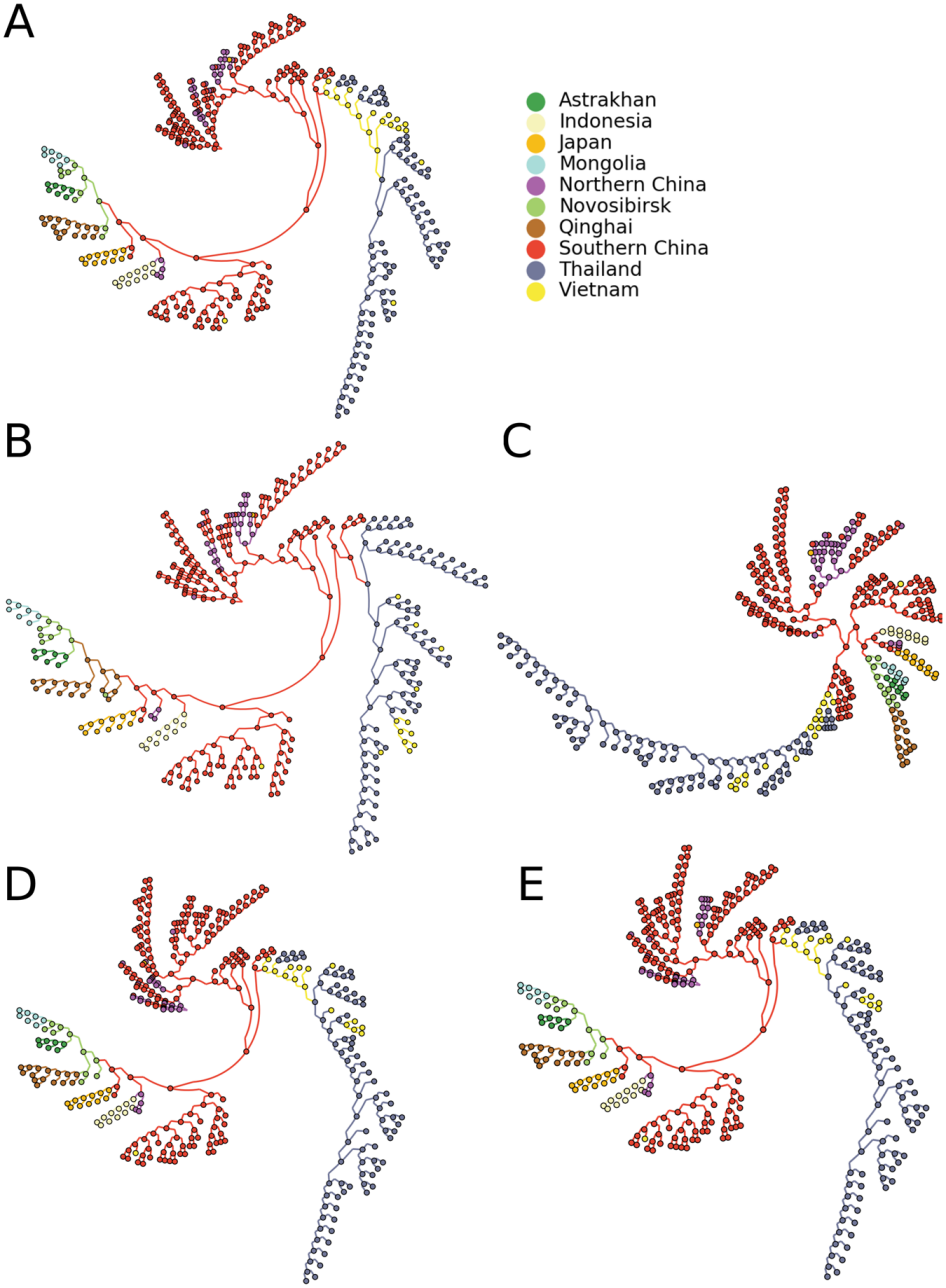
Phylogenetic trees with locations mapped to internal nodes and branches as indicated by the colors. The trees were generated by maximum parsimony (**A**) neighbor joining (**B**) UPGMA (**C**) maximum likelihood using the Jukes-Cantor model (**D**) and maximum likelihood using the GTR model (**E**). Ancestral states were inferred by parsimony. The visualization was performed using GraPhlAn^34^.

H5N1 influenza viruses are circulating in birds, including domestic poultry and waterfowl^23,24^. Occasionally humans can be infected, resulting in severe disease and high mortality rates, making H5N1 influenza a pandemic threat although it is currently not easily transmitted between humans^25^. For this reason, studying the origin and spread of this virus is essential. Using five different reconstruction methods, we inferred phylogenetic trees on 190 HA sequences from H5N1 viruses isolated between 1996 and 2005 across Asia and Europe (Fig. 2). All methods result in different tree topologies. This was confirmed by calculating the Robinson-Foulds (RF) metric (Table 1), which measures distances between phylogenetic trees^26^. The trees generated by maximum likelihood using either the Jukes-Cantor or the general time reversible model were most similar to each other, with a Robinson-Foulds distance of 28, while all other trees differed to a greater degree, with distances between 144 and 242. To evaluate which distances deviated how far from the mean, we calculated the corresponding z-scores for all distances by subtracting the mean and dividing by the standard deviation (Table 1). In addition to the comparison between the parsimony and UPGMA tree, all comparisons with the NJ tree resulted in positive z-scores, indicating that this tree differed the most compared to the others. However, the z-scores were generally low. To reconstruct migration patterns, we inferred geographical locations for internal nodes using parsimony ASR (as indicated by colors in Fig. 2) using the clustered locations. For the parsimony, UPGMA and NJ tree, the root is placed in Southern China, while Northern China is inferred for the ML trees. Southern China is also mainly inferred on the trunk of all trees, indicating that the virus originated from this region and outbreaks in other locations are seeded from there, which is in line with previous analyses^1,16^. On many branches, the migration patterns along the trees are similar, e.g. with links from Novosibirsk (light green) to Mongolia (light blue) and Astrakhan (dark green). However, different routes of migration can be seen in each tree.

**Table 1:** Pairwise Robinson Foulds metric for all five inferred trees (below the main diagonal) and the corresponding z-scores (above the main diagonal).

	UPGMA	NJ	Parsimony	MLJC	MLGTR
UPGMA	0	1.38	0.60	−0.14	−0.14
NJ	242	0	0.22	0.57	0.57
Parsimony	198	176	0	−0.31	−0.35
MLJC	156	196	146	0	−2.40
MLGTR	156	196	144	28	0

**Figure 3:**
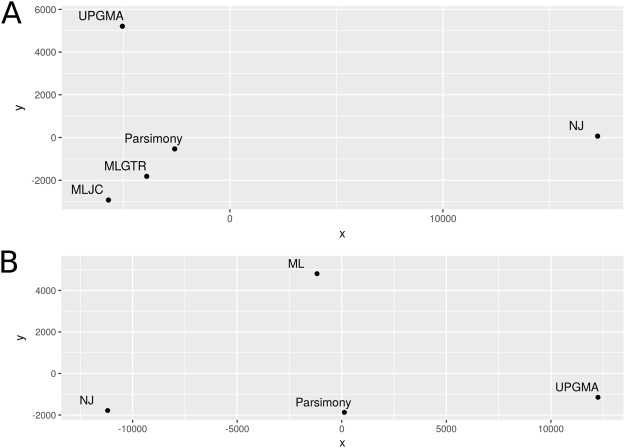
Visualization of pairwise Fréchet tree distances. A nonmetric multidimensional scaling was performed on the pairwise Fréchet tree distances to plot the differences in a 2-dimensional space. (**A**) Fréchet tree distances using parsimony for ancestral reconstruction. (**B**) Fréchet tree distances using maximum likelihood for ancestral reconstruction.

To assess the differences between these spread patterns, we calculated the discrete Fréchet tree distance between the trees (Table 2). The results describe how far the spread patterns along the phylogenetic trees deviate. As already indicated by the RF metric, the spread patterns inferred on the two trees reconstructed using maximum likelihood are most similar to each other, with a distance of 573 (Table 2). Otherwise, the distances between most pairs ranged from around 6,000 to 8,000. A striking exception is the tree inferred using neighbor joining, with distances between 20,000 to the parsimony tree and 23,000 to the MLJC tree. The pairwise distances were plotted after performing a multidimensional scaling (Fig. 3A), which underlines that the phylogeographic spread inferred on the neighbor joining tree deviates from all other trees, which show more similar geographic spread patterns. The plot shows a tight clustering of the ASRs on the parsimony and ML trees, although this is not directly observed in the distances and is thus likely introduced as an artifact by the multidimensional scaling visualization.The similarity between the MLJC and MLGTR tree is not surprising, considering the small RF distance (Table 1). For the NJ tree, the discrete Fréchet tree distances agree with the trend already shown in the RF distance as well, but the difference is more pronounced. We calculated z-scores for the Fréchet tree distances (Table 2) and only the comparisons to the NJ tree show positive scores. These z-scores are mainly larger compared to the z-scores calculated for the RF distances, meaning that the Fŕechet tree distances for the NJ tree deviate more from the sample mean than the RF distances. In summary, the discrete Fréchet distances generally correlate to the RF distances, but some differences are a lot more distinct, as shown for the NJ tree using z-scores and multidimensional scaling. This indicates that even small differences in the tree can have larger effects on phylogeographic reconstructions on certain topologies.

**Table 2:** Pairwise Fréchet tree distances for all five inferred trees using parsimony for the ancestral character state reconstruction (below the main diagonal) and the corresponding z-scores (above the main diagonal).

	UPGMA	NJ	Parsimony	MLJC	MLGTR
UPGMA	0	1.24	−0.52	−0.51	−0.63
NJ	22905.86	0	0.94	1.27	1.04
Parsimony	8043.998	20399.78	0	−0.68	−0.75
MLJC	8132.359	23165.58	6678.068	0	−1.34
MLGTR	7052.893	21257.84	6050.793	573.3009	0

To check which spread routes cause the large discrepancy between the NJ tree and all others, we checked which nodes in the NJ tree have the highest distances to the other trees after correcting for the number of paths. In all cases, two nodes contribute the most to the overall distance: the first internal node with Thailand assigned as its location and the first internal node with Qinghai assigned. The NJ tree infers a migration route from Southern China to Thailand and from Southern China to Qinghai, while all other trees infer a route from Vietnam to Thailand and from Novosibirsk to Qinghai. Due to the relatively large geographical distances between these paths, the trees show a high discrete Fréchet distance. This shows how topological differences in badly resolved branches can substantially influence the inferred phylogeographic spread. Using the Fréchet distance, our method provides a way to easily assess these differences across different phylogenetic trees.

We further investigated whether this observed variability was due to the use of parsimony for the ancestral state reconstruction. We therefore additionally performed maximum likelihood ASR on all five trees. The reconstructed phylogeographic spread was visualized on the phylogenetic trees (Supplementary Fig. 1).

Using maximum likelihood for ASR resulted in a different phylogeographic reconstruction for each tree compared to the parsimony reconstruction (Supplementary Table 1). Between 3 (UPGMA tree) and 12 (Parsimony and MLGTR tree) internal nodes had different character states assigned (between 1.6% and 6.3%), while the discrete Fréchet tree distances ranged roughly from 2,100 to 10,000. When comparing the discrete Fréchet tree distances between the five maximum likelihood ASRs, we still observe considerable variation between the spread patterns, although the results are notably different from the parsimony ASR (Table 3). Only the comparison between the UPGMA and NJ trees still shows a high Fréchet tree distance of around 23,000, comparable to the result using parsimony ASR. All other distances involving the NJ tree were distinctly lower than before. Instead, the second highest distances are found between the UPGMA and both ML trees. The ML trees are most similar with a distance of 0, showing that despite of topological differences, the paths along two trees can be equal. In the resulting multidimensional scaling plot (Fig. 3B), NJ is no longer a clear outlier to all other trees and instead, both NJ and UPGMA show similar distances to ML and parsimony. Overall, these results imply that both the method of tree inference as well as the one for ASR may substantially affect the differences between reconstructed phylogeographic spread patterns.

**Table 3:** Pairwise Fréchet tree distances for all five inferred trees using maximum likelihood for the ancestral character state reconstruction (below the main diagonal) and the corresponding z-scores (above the main diagonal).

	UPGMA	NJ	Parsimony	MLJC	MLGTR
UPGMA	0	1.99	−0.23	0.64	0.61
NJ	23451.97	0	−0.46	0.21	0.47
Parsimony	8486.976	6949.106	0	−0.87	−0.87
MLJC	14317.09	11452.11	4145.878	0	−1.49
MLGTR	14113.19	13171.35	4145.878	0	0

## Discussion

By using the discrete Fréchet distance on phylogenetic trees, we here present a distance measure to quantify geographical differences between different phylogeographic reconstructions. Our method is flexible since it can compare geographic spread patterns across different tree topologies and only the leaf nodes, i.e. the data used to reconstruct the trees, should be the same. The applicability on different topologies is achieved by comparing paths to each other instead of pairwise comparisons between nodes, which is usually done when ancestral character state reconstructions are evaluated^7–10^. Since our method is looking at paths, trees need to be rooted e.g. by using an outgroup. We can then define a path of locations from the root, representing the origin of the species, to the observed data on the leaf nodes.

With this method, different phylogeographic reconstructions can be compared to each other. To demonstrate a possible application, we here performed a phylogeographic reconstruction on phylogenetic trees inferred with different methods to assess the effect of tree topology on inferred spread paths. For this question, we analyzed a dataset of 190 HA sequences of H5N1 influenza viruses and conclude that topological differences influence phylogeographic spread patterns in different ways. The result is further dependent on the method used for ancestral state reconstruction. We first used parsimony to reconstruct ancestral states. The trees inferred using maximum likelihood (MLJC and MLGTR) are similar in topology with a small Robinson-Foulds distance and small differences in geographic spread, as confirmed with the discrete Fréchet tree distance. In comparison, the NJ tree shows large discrete Fréchet tree distances to all other trees, although its Robinson-Foulds distance is only slightly larger compared to distances between the other trees. Small differences in tree inference can therefore have large effects on phylogeographic spread, if badly resolved branches contain locations with large geographical distances. However, these discrepancies were in parts specific to the method used for ancestral state reconstruction. Reconstruction using maximum likelihood still resulted in large differences in phylogeographic spread patterns on the different topologies, with exception of the ML trees, but other pairs of trees showed the largest Fréchet tree distances. Overall, we conclude that both tree topology and ancestral state reconstruction result in variation in phylogeographic spread paths along trees. Which reconstruction reflects the actual origin and spread most closely cannot be deduced from this analysis alone, however. For this, a reference needs to be available so that the tree with the smallest discrete Fréchet tree distance to the reference can be determined.

Another relevant application could be to compare phylogeographic reconstructions generated by different methods. Currently, phylogeographic methods are not frequently compared to each other. For this, suitable references need to be available. Recent outbreaks like the pandemic H1N1 influenza virus in 2009 have been studied in detail and provide information about possible transmission patterns, aiding both the evaluation of phylogeographic methods and the evaluation of spread simulations. Sophisticated disease spread simulations have been developed in recent years. Many combine mathematical or agent-based models with population as well as mobility data to simulate the outbreak and migration of a disease^27–31^. By comparing these models to observed data, i.e. from the 2009 influenza pandemic, it has been shown that simulations accurately predict the peak activity in single countries^32^. By combining simulated geographical spread with phylogenetic information, it will be possible to generate a reference suitable for evaluation with the discrete Fréchet tree distance on phylogenetic trees. We here have provided a proof of concept that this distance measure can be applied to evaluate different phylogeographic spread patterns across phylogenetic trees and therefore enables a comprehensive benchmark of different phylogeographic reconstruction methods. It further allows to assess the variance within one method, e.g. by comparing spread patterns inferred using different parameters, or by evaluating a large number of plausible solutions, like the set of trees in the posterior sample generated by a BEAST analysis. These studies would help to assess the robustness of a reconstruction method and aid the interpretation of phylogeographic reconstructions.

## Electronic supplementary material

Supplementary Information

## Data Availability

All data and software necessary to reproduce the analysis in this paper and to apply the discrete Fréchet distance on phylogenetic trees on different datasets are available at https://github.com/hzi-bifo/FrechetTreeDistance^33^.

## References

[1] Lemey P, Rambaut A, Drummond AJ, Suchard MA (2009). Bayesian phylogeography finds its roots. PLoS Comput. Biol..

[2] Lemey P, Rambaut A, Welch JJ, Suchard MA (2010). Phylogeography takes a relaxed random walk in continuous space and time. Mol. Biol. Evol..

[3] Bouckaert R (2016). Phylogeography by diffusion on a sphere: whole world phylogeography. PeerJ.

[4] Bedford T (2015). Global circulation patterns of seasonal influenza viruses vary with antigenic drift. Nature.

[5] Lemey P (2014). Unifying viral genetics and human transportation data to predict the global transmission dynamics of human influenza H3N2. PLoS Pathog..

[6] Faria NR (2014). HIV epidemiology. The early spread and epidemic ignition of HIV-1 in human populations. Science.

[7] Martins EP (1999). Estimation of ancestral states of continuous characters: a computer simulation study. Systematic Biology.

[8] Royer-Carenzi M, Didier G (2016). A comparison of ancestral state reconstruction methods for quantitative characters. J. Theor. Biol..

[9] Oakley TH, Cunningham CW (2000). Independent contrasts succeed where ancestor reconstruction fails in a known bacteriophage phylogeny. Evolution.

[10] Webster AJ, Purvis A (2002). Testing the accuracy of methods for reconstructing ancestral states of continuous characters. Proc. Biol. Sci..

[11] Hanson-Smith V, Kolaczkowski B, Thornton JW (2010). Robustness of ancestral sequence reconstruction to phylogenetic uncertainty. Mol. Biol. Evol..

[12] Fréchet M (1906). Sur quelques points du calcul fonctionnel. Rendiconti del Circolo Mathematico di Palermo.

[13] Eiter, T. & Mannila, H. Computing Discrete Fréchet Distance. Tech. Report CD-TR 94/64, *Christian Doppler Laboratory for Expert Systems, TU Vienna, Austria* (1994).

[14] Brockmann D, Helbing D (2013). The hidden geometry of complex, network-driven contagion phenomena. Science.

[15] Bao Y (2008). The influenza virus resource at the National Center for Biotechnology Information. J. Virol..

[16] Wallace RG, Hodac H, Lathrop RH, Fitch WM (2007). A statistical phylogeography of influenza A H5N1. Proc Natl Acad Sci USA.

[17] Edgar RC (2004). MUSCLE: multiple sequence alignment with high accuracy and high throughput. Nucleic Acids Res..

[18] Capella-Gutiérrez S, Silla-Martínez JM, Gabaldón T (2009). trimAl: a tool for automated alignment trimming in large-scale phylogenetic analyses. Bioinformatics.

[19] Schliep KP (2011). phangorn: phylogenetic analysis in R. Bioinformatics.

[20] Paradis, E. & Schliep, K. ape 5.0: an environment for modern phylogenetics and evolutionary analyses in R. *Bioinformatics* (2018). 10.1093/bioinformatics/bty633 30016406

[21] Kahle, D. & Wickham, H. ggmap: Spatial Visualization withggplot2. *The R Journal* (2013).

[22] Hijmans, R. J. *geosphere: Spherical Trigonometr*y. (2017).

[23] Chen H (2005). Avian flu: H5N1 virus outbreak in migratory waterfowl. Nature.

[24] Li KS (2004). Genesis of a highly pathogenic and potentially pandemic H5N1 influenza virus in eastern Asia. Nature.

[25] Lai S (2016). Global epidemiology of avian influenza A H5N1 virus infection in humans, 1997-2015: a systematic review of individual case data. Lancet Infect. Dis..

[26] Robinson DF, Foulds LR (1981). Comparison of phylogenetic trees. Math. Biosci..

[27] Van den Broeck W (2011). The GLEaMviz computational tool, a publicly available software to explore realistic epidemic spreading scenarios at the global scale. BMC Infect. Dis..

[28] Balcan D (2010). Modeling the spatial spread of infectious diseases: the GLobal Epidemic and Mobility computational model. J. Comput. Sci..

[29] Ferguson NM (2006). Strategies for mitigating an influenza pandemic. Nature.

[30] Hufnagel L, Brockmann D, Geisel T (2004). Forecast and control of epidemics in a globalized world. Proc Natl Acad Sci USA.

[31] Eubank S (2004). Modelling disease outbreaks in realistic urban social networks. Nature.

[32] Tizzoni M (2012). Real-time numerical forecast of global epidemic spreading: case study of 2009 A/H1N1pdm. BMC Med..

[33] Reimering, S., Munoz, S. & McHardy, A. C. hzi-bifo/FrechetTreeDistance: Frechet Tree Distance. *Zenodo*, 10.5281/zenodo.1460594 (2018).

[34] Asnicar F, Weingart G, Tickle TL, Huttenhower C, Segata N (2015). Compact graphical representation of phylogenetic data and metadata with GraPhlAn. PeerJ.

