# Supplementary material for: A Fréchet tree distance measure to compare phylogeographic spread paths across trees
Source: Sci Rep. 2018 Nov 19;8:17000. doi: 10.1038/s41598-018-35421-4 (PMC6242967; doi:10.1038/s41598-018-35421-4)
Supplement: Supplementary file 1 — Supplementary Information [file 41598_2018_35421_MOESM1_ESM.pdf]

A Fréchet tree distance measure to compare phylogeographic spread paths across trees

Susanne Reimering[1], Sebastian Muñoz[1], Alice C. McHardy[1,2 *]

[1]Department for Computational Biology of Infection Research, Helmholtz Center for Infection Research, Braunschweig, Germany
[2]German Center for Infection Research (DZIF), Braunschweig, Germany

*Correspondence to: Alice.McHardy@helmholtz-hzi

Supplementary Figure 1

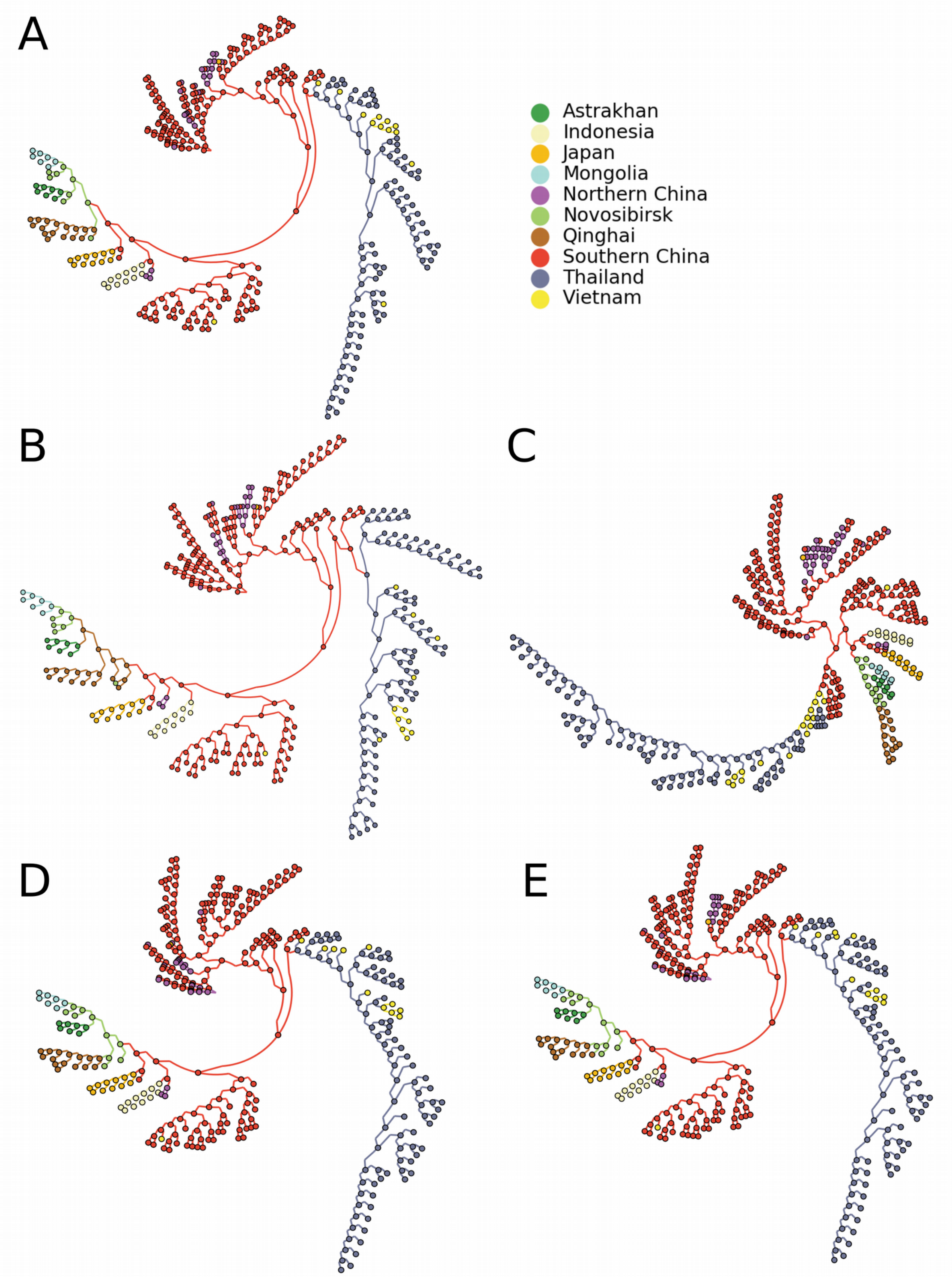

Supplementary Figure 1: Phylogenetic trees with locations inferred by maximum likelihood and mapped to internal nodes and branches as indicated by the colors. The trees were generated by maximum parsimony (A), neighbor joining (B), UPGMA (C), maximum likelihood using the Jukes-Cantor model (D) and maximum likelihood using the GTR model (E) on the dataset with clustered locations.

Supplementary Table 1

	Fréchet tree distance	Number of nodes with different locations	Amount of nodes with different locations
UPGMA	2174.67	3	0.016
NJ	4509.48	4	0.021
Parsimony	10000.33	12	0.063
MLJC	7917.40	10	0.053
MLGTR	8204.06	12	0.063

Supplementary Table 1: Comparison between phylogeographic spread inferred using parsimony and maximum likelihood on the same tree topologies
